# Functional Complementarity of Arbuscular Mycorrhizal Fungi and Associated Microbiota: The Challenge of Translational Research

**DOI:** 10.3389/fpls.2018.01407

**Published:** 2018-09-24

**Authors:** Alessandra Turrini, Luciano Avio, Manuela Giovannetti, Monica Agnolucci

**Affiliations:** Department of Agriculture, Food and Environment, University of Pisa, Pisa, Italy

**Keywords:** arbuscular mycorrhizal fungi, mycorrhizosphere, beneficial soil bacteria, plant growth promoting bacteria, mycorrhiza helper bacteria, P solubilization

## Arbuscular mycorrhizal fungi and the associated microbiota

One of the main challenge for humanity in the years to come is represented by the production of enough food for a growing global population, while reducing the use of pesticides and chemical fertilizers and maintaining environmental quality and natural resources for future generations. The new paradigm in agriculture—sustainable intensification—may be implemented by the efficient use of practices enhancing the activity of beneficial soil microorganisms, essential elements of soil nutrient flows, at the basis of long-term soil productivity and health. There is a growing awareness of the essential roles played by soil microorganisms in human nutrition and welfare and of the economic importance of ecosystem services they provide in agriculture, forestry, and society (Philippot et al., 2013; Avio et al., 2018).

Among beneficial microorganisms, arbuscular mycorrhizal (AM) fungi (AMF) have been long known for their positive impact on plant growth and health. AMF are obligate biotrophs, establishing mutualistic symbioses with the roots of the majority of land plants, including the most important food crops, such as cereals, pulses, potatoes, fruit trees, vegetables, and medicinal plants. They obtain carbon from the host plants, and, in exchange, facilitate the uptake and transfer of mineral nutrients—phosphorus (P), nitrogen (N), sulfur (S), potassium (K), calcium (Ca), copper (Cu), and zinc (Zn)—from the soil, by means of an extensive network of hyphae spreading from colonized roots into the surrounding environment. Beyond improving mineral nutrition, AMF increase plant tolerance to biotic and abiotic stresses, and provide multiple ecosystem services in natural and agricultural environments, from the completion of biogeochemical cycles to the maintenance of biological soil fertility (Smith and Read, 2008). In addition to such multifunctional roles, AMF induce changes in plant secondary metabolism, stimulating the biosynthesis of phytochemical compounds, such as polyphenols and carotenoids, thus leading to the production of safe and high-quality foods, able to promote human health (Sbrana et al., 2014).

A number of multimodal investigations showed the occurrence of diverse assemblages of bacterial communities living strictly associated with AMF spores, extraradical mycelium and mycorrhizal roots, in the mycorrhizosphere. The use of transmission electron microscope allowed the detection of unculturable endobacteria inside the spores of some AMF species (Mosse, 1970; Bianciotto et al., 1996) and of free living bacteria embedded in the spore wall layers or in the microniches formed by the peridial hyphae interwoven around the spores in the sporocarps (Ames et al., 1989; Filippi et al., 1998). Molecular studies confirmed the occurrence of either Mollicutes-related and unculturable endosymbionts (Desirò et al., 2014) or bacteria strictly associated with the spores of different AMF taxa, affiliated with *Cellvibrio, Chondromyces, Flexibacter, Lysobacter*, and *Pseudomonas* (Roesti et al., 2005), Proteobacteria and Actinobacteria (Long et al., 2008), and Actinomycetales, Bacillales, Rhizobiales, Pseudomonadales, Burkholderiales, including *Arthrobacter, Streptomyces, Bacillus, Paenibacillus, Pseudomonas, Herbaspirillum, Massilia, Rhizobium*, and *Sinorhizobium* (Agnolucci et al., 2015).

Overall, the ultrastructural and molecular studies revealed the complexity and diversity of bacterial communities living associated with AMF, suggesting putative important functional roles as plant growth promoting (PGP) bacteria and mycorrhiza helper bacteria (MHB), able to promote AMF activity and development. However, in order to verify such hypothesis and to unravel the physiological interactions between AMF and associated bacteria possibly leading to positive synergistic effects on plant nutrition and health, it is crucial to isolate the bacteria in pure culture. Using culture-dependent approaches many authors obtained a number of bacterial strains from the sporosphere of *Glomus versiforme* and *Glomus clarum* NT4 (Mayo et al., 1986; Xavier and Germida, 2003), *Gigaspora margarita* (Cruz et al., 2008), *Glomus mosseae*, and *Glomus intraradices* (syn. *Rhizophagus intraradices*) (Bharadwaj et al., 2008b; Battini et al., 2016b), most of which were further studied for PGP activities, with the aim of their possible utilization, either as individual strains or consortia, as biocontrol agents, biofertilisers, and bioenhancers (Rouphael et al., 2015).

## A network of functional interactions among AMF and associated microbiota

As early as 1959 soil bacteria were studied for their ability to affect AMF spore germination and hyphal growth (Mosse, 1959). Subsequent works confirmed their functional role in the promotion of mycorrhizal activity (Azcón, 1989), and proposed the term “mycorrhiza helpers” for such bacteria (Frey-Klett et al., 2007). Many actinobacteria isolated from the soil, in particular species belonging to the genera *Streptomyces* and *Corynebacterium*, as well as species of *Pseudomonas* increased the germination of *G. versiforme, G. mosseae*, and *G. margarita* spores (Mayo et al., 1986; Tylka et al., 1991; Carpenter-Boggs et al., 1995), while *Klebsiella pneumoniae* and *Trichoderma* sp. enhanced germlings growth in *Glomus deserticola* and *G. mosseae* (Will and Sylvia, 1990; Calvet et al., 1992). Bacteria isolated from the mycorrhizosphere, either from mycorrhizal roots or from AMF spores and hyphae, were able to enhance spore germination, germling growth, and AMF root colonization (Mayo et al., 1986; Xavier and Germida, 2003; Giovannetti et al., 2010). One of the possible mechanistic explanations of the phenomenon is based on the ability of several bacteria to decompose insoluble biopolymers like chitin and chitosan, the two main constituents of AMF spore walls, thus boosting germination. Such hypothesis is supported by the frequent isolation of chitinolytic bacteria from spores of *Glomus macrocarpum, G. mosseae*, and *R. intraradices* (Ames et al., 1989; Filippi et al., 1998; Battini et al., 2016b). Mycorrhizosphere and sporosphere bacteria may act as “mycorrhiza helper” also by improving the growth of extraradical mycelium (ERM), the fine absorbing network of hyphae extending around the roots. *In vitro* studies showed that ERM length of *G. intraradices* and *Rhizophagus irregularis* was increased by *Paenibacillus rhizosphaerae, Rhizobium etli*, and several strains of *Azospirillum* and *Pseudomonas* (Bidondo et al., 2011; Ordoñez et al., 2016). Accordingly, *in vivo* investigations confirmed large ERM growth, boosted by *Pseudomonas fluorescens, Burkholderia cepacia, Sinorhizobium meliloti*, and *Streptomyces* spp. in *Glomus caledonium, G. intraradices*, and *R. irregularis* (Ravnskov and Jakobsen, 1999; Battini et al., 2017). Moreover, a number of indole acetic acid-producing bacteria were isolated from *G. mosseae* and *R. irregularis* spores, including several actinobacteria species, *Paenibacillus favisporus, S. meliloti*, and *Fictibacillus barbaricus* (Bidondo et al., 2011; Battini et al., 2017). However, the mechanistic explanation of ERM development promotion remains to be investigated.

Beyond “mycorrhizal helper” activity, AMF associated microbiota is fundamental for the maintenance of plant health, as it can protect plants from soilborne diseases and abiotic stresses. Actually, some strains were able to inhibit the growth of fungal pathogens by either producing antibiotics (Li et al., 2007; Bharadwaj et al., 2008a) or competing for iron nutrition by secreting siderophores, high-affinity iron-chelating compounds able to bind soluble Fe^3+^ (Whipps, 2001; Battini et al., 2016b). Interestingly, the AMF associated bacterial strains *Massilia* sp. RK4 and *Pseudomonas koreensis* S2CB35 were reported to improve maize tolerance to salinity (Krishnamoorthy et al., 2016; Selvakumar et al., 2018).

Another essential role played by AMF associated microbiota is represented by the solubilization of P, a key mineral nutrient which is poorly available to plants in most agricultural soils, as the result of its immobilization and precipitation with other soil minerals—iron and aluminum in acid and calcium in alkaline soils. P-mobilizing bacteria were isolated from AMF spores of *G. mosseae* and *R. intraradices*, and were ascribed to *Streptomyces* and *Leifsonia* species (Mohandas et al., 2013) and *Streptomyces* spp., *Bacillus pumilus, Lisinobacillus fusiformis*, and *S. meliloti* (Battini et al., 2016b). Such bacteria represent a sustainable strategy for the mobilization of the soil P pool and the facilitation of P uptake by mycorrhizal plants (Battini et al., 2017).

Also the acquisition of N, a major plant nutrient, may be mediated by AMF associated bacteria, as different species of the N-fixers *Rhizobium* and *Sinorhizobium*, isolated from AMF spores (Bharadwaj et al., 2008a; Agnolucci et al., 2015; Battini et al., 2016b), promoted mycorrhizal functioning, and plant mineral nutrition (Battini et al., 2017). Moreover, in recent years evidence suggested that bacteria isolated from AMF spores may improve the concentration of the health-promoting compound rosmarinic acid in basil plants, by modulating the expression of genes involved into its biosynthesis (Battini et al., 2016a).

The multifunctional activities of AMF associated microbiota described so far clearly show the complex and previously unimagined network of interactions involving AMF and their associated microbiota, that may encompass not univocal outcomes, depending on the identity of the taxa active in the mycorrhizosphere. In order to exploit the functional complementarity of the diverse AMF-bacteria combinations, extensive studies should be carried out to answer basic questions concerning not only the isolation and identification of bacterial strains involved in specific functional activities, but also the spatio-temporal and environmental conditions affecting their behavior and its mechanistic explanation.

## The challenge of translational research

One of the main problems to be tackled when trying to translate the findings of fundamental research and laboratory studies into new tools and meaningful innovations in agricultural practices is represented by the absence of a systematic collection of data enabling scientists to gather information on the source of AMF associated beneficial bacteria, i.e., the different AMF genera, species and strains and where they were isolated from. Indeed, most data were obtained using AMF whose taxonomy was repeatedly and widely changed, emended and subverted in recent years (Oehl et al., 2011; Redecker et al., 2013; Sieverding et al., 2014). As an example, the ubiquitous species *F. mosseae* was named *Endogone mosseae* in 1968 and *G. mosseae* in 1974, while *Racocetra coralloidea* was known as *Gigaspora coralloidea* since 1974 and as *Scutellospora coralloidea* since 1986. As long known AMF species and genera were renamed, it is very difficult to link the taxon currently under investigation with its very properties, including the diverse spore-associated bacterial communities, whose composition differs in the diverse AMF isolates (Agnolucci et al., 2015).

In addition, very rarely scientists named the strains used in their experiments and deposited them in a germplasm bank, thus hindering the possibility of retrieving both AMF and the relevant associated microbiota for further investigations on their functional properties. On the other hand, such strains were not elite strains, i.e., the result of a fine tuned selection aimed at detecting the best performing ones, but just those able to produce, in pot-cultures, large quantities of mycelium, and spores from which bacteria could be easily isolated. Thus, the great majority of AMF are still to be studied as the home and source of beneficial bacteria. Such flaws are currently mirrored by the low number of AMF genotypes utilized as inoculants available on the market globally, and by the lack of innovative products or new formulations, developed from the isolation of AMF associated bacterial strains, selected for their specific activities to be applied as single strains or combined consortia. Moreover, the efficacy of the available AMF and associated bacteria should be deeply studied after inoculation in the field.

Another major problem that hindered the understanding of the diverse properties of mycorrhizospheric microbiota and its functional complementarity with AMF, leading to the promotion of mycorrhizal activity and host plant performance, is represented by the lack of studies on gene expression changes regulated by the presence of AMF associated bacterial isolates. Actually, while transcriptome studies, recently carried out by RNAseq technology, obtained the expression profiles of genes specifically regulated in mycorrhizal model and crop plants (Handa et al., 2015; Vangelisti et al., 2018), only few data are available on the expression levels of transcripts modulated by AMF associated beneficial bacteria (Battini et al., 2016a). Functional genomics studies are needed in order to advance our understanding of the multifunctionality and complementarity of AMF associated microbiota, and to boost translational research on the selection of the best performing strains and consortia, showing the most desirable functional activities relevant for plant growth, nutrition and health. Bridging the gap between basic science and the formulation of innovative products for the sustainable intensification of food production systems, represents the real next challenge in the years to come.

## Author contributions

All authors listed have made a substantial, direct and intellectual contribution to the work, and approved it for publication.

### Conflict of interest statement

The authors declare that the research was conducted in the absence of any commercial or financial relationships that could be construed as a potential conflict of interest.
